# Polyethylene Glycol-Mediated Directional Conjugation of Biological Molecules for Enhanced Immunoassays at the Point-of-Care

**DOI:** 10.3390/polym15153316

**Published:** 2023-08-05

**Authors:** Dheerendranath Battalapalli, Purbali Chakraborty, Disha Jain, Stephen K. Obaro, Umut A. Gurkan, Robert A. Bonomo, Mohamed S. Draz

**Affiliations:** 1 Department of Medicine, Case Western Reserve University School of Medicine, Cleveland, OH 44106, USA; 2 Division of Pediatric Infectious Diseases, Department of Pediatrics, University of Alabama at Birmingham, Birmingham, AL 35233, USA; 3 Mechanical and Aerospace Engineering Department, Case Western Reserve University, Cleveland, OH 44106, USA; 4 Department of Biomedical Engineering, Case Western Reserve University, Cleveland, OH 44106, USA; 5 Case Comprehensive Cancer Center, Case Western Reserve University, Cleveland, OH 44106, USA; 6 Research Service, Louis Stokes Cleveland Department of Veterans Affairs Medical Center, Cleveland, OH 44106, USA; 7 Department of Molecular Biology and Microbiology, Case Western Reserve University School of Medicine, Cleveland, OH 44106, USA; 8 CWRU-Cleveland VAMC Center for Antimicrobial Resistance and Epidemiology, Cleveland, OH 44106, USA; 9 Department of Biomedical Engineering, Cleveland Clinic, Cleveland, OH 44106, USA

**Keywords:** biological molecules, antibody, directional, site-specific, controlled, conjugation, immunoassay, screen-printed electrodes, point of care

## Abstract

Rapid and reliable point-of-care (POC) diagnostic tests can have a significant impact on global health. One of the most common approaches for developing POC systems is the use of target-specific biomolecules. However, the conjugation of biomolecules can result in decreased activity, which may compromise the analytical performance and accuracy of the developed systems. To overcome this challenge, we present a polymer-based cross-linking protocol for controlled and directed conjugation of biological molecules. Our protocol utilizes a bifunctional thiol-polyethylene glycol (PEG)-hydrazide polymer to enable site-directed conjugation of IgG antibodies to the surface of screen-printed metal electrodes. The metal surface of the electrodes is first modified with thiolated PEG molecules, leaving the hydrazide groups available to react with the aldehyde group in the Fc fragments of the oxidized IgG antibodies. Using anti-*Klebsiella pneumoniae* carbapenemase-2 (KPC-2) antibody as a model antibody used for antimicrobial resistance (AMR) testing, our results demonstrate a ~10-fold increase in antibody coupling compared with the standard *N*-hydroxysuccinimide (NHS)-based conjugation chemistry and effective capture (>94%) of the target KPC-2 enzyme antigen on the surface of modified electrodes. This straightforward and easy-to-perform strategy of site-directed antibody conjugation can be engineered for coupling other protein- and non-protein-based biological molecules commonly used in POC testing and development, thus enhancing the potential for improved diagnostic accuracy and performance.

## 1. Introduction

The demand for point-of-care (POC) medicine is rapidly increasing in our modernized and globalized world. POC diagnostics have revolutionized healthcare by providing rapid and accurate disease diagnosis at the patient’s bedside or in a clinical setting [1]. Immunoassays are widely used in POC diagnostics for detecting analytes such as proteins, nucleic acids, and small molecules [2]. However, the sensitivity and specificity of immunoassays are limited by the availability and orientation of the antibodies on the surface of the assay platform [3]. Conventional conjugation methods involve random and non-specific chemical reactions between the antibody and the conjugation partner, such as *N*-hydroxysuccinimide (NHS) ester or maleimide chemistry, which can result in low yields, heterogeneous products, and reduced antibody activity [4,5]. Site-specific modification strategies have been developed to overcome limitations by enabling the selective conjugation of antibodies to specific sites and improving the availability and orientation of the antibodies on the surface of the assay platform [6,7,8,9,10,11,12,13].

Chemical conjugation using functional polymers such as polyethylene glycol (PEG) has emerged as a superior alternative to traditional conjugation methods [14]. PEG can be easily conjugated to antibodies through various chemistries and offers several advantages compared to other polymers, including high water solubility, low toxicity, and low immunogenicity [15,16,17]. Moreover, PEG conjugation can enhance the stability and shelf-life of the antibody, increase the solubility and bioavailability of the conjugate, and reduce the clearance rate from the body [18,19,20]. These properties make PEG an ideal candidate for conjugation with antibodies in POC diagnostics.

Point-of-care diagnostics have been a game-changer in infectious disease control, providing rapid, patient-centric testing with immediate results. The importance of such rapid diagnostic tools has been highlighted in recent global health crises, such as the COVID-19 pandemic. PEGylated antibodies have been employed in lateral flow immunoassays (LFIs) for the detection of SARS-CoV-2 antigens [21]. The stability of these PEGylated antibodies allows the LFIs to deliver rapid, point-of-care diagnostics in diverse settings, potentially contributing to widespread pandemic control. The PEGylation process has proved particularly beneficial for the detection of antigens that are usually present at low concentrations in the early stages of infection, making them challenging for traditional immunoassays to identify. PEGylated antibodies, due to their enhanced stability and activity, can bind to these low-concentration antigens and generate a stronger and more detectable signal [22]. This increased sensitivity and specificity offered by PEGylated antibodies plays a critical role in early disease detection, leading to timely treatment and better patient outcomes.

Furthermore, PEGylated antibodies have found utility in the detection of bacterial infections. The use of PEGylated antibodies in LFIs has been effective in diagnosing infections caused by MRSA, Tuberculosis (TB), streptococcus pneumoniae and staphylococcus aureus [23,24,25,26]. By improving the detection limit and specificity of these immunoassays, PEGylated antibodies have bolstered the clinical utility of these diagnostic tools. PEGylation also plays a crucial role in enhancing the stability of biosensors used for infectious disease diagnosis. For instance, PEGylated antibodies have been immobilized on the surface of electrochemical biosensors, leading to a significant improvement in the detection of the hepatitis B surface agent [27]. PEGylation reduced non-specific binding, thereby improving the biosensor’s sensitivity and reliability.

One of the major advantages of chemical conjugation using functional polymers like PEG is that it enables site-specific modification for antibody conjugation [28,29,30]. These approaches have gained significant attention in the development of POC diagnostics. PEG enables the selective and controlled conjugation of antibodies to specific sites, reducing the risk of antibody denaturation and loss of function [31]. Moreover, PEG conjugated antibodies offer enhanced performance and accuracy in immunoassays by improving the availability and orientation of the antibodies on the assay platform.

Here, we present a novel approach for PEG-mediated site-specific antibody conjugation for the development of a POC-based electrode microchip immunoassay. We demonstrate the efficacy of our approach by developing anti-Klebsiella pneumoniae carbapenemase-2 (KPC-2) antibody-modified screen-printed gold (Au) electrodes with improved conjugation and target-capture efficiency compared to conventional conjugation methods. In our approach, a heterobifunctional linker of thiol-PEG-hydrazide (SH-PEG-hydrazide) was used to modify the carbohydrate residues of the Fc region of the polyclonal anti-KPC-2 IgG antibody. The antibody modified with PEG-thiol is then allowed to react with the surface of Au electrodes via the well-known thiol-metal bonding chemistry. Our approach represents a significant advancement in the development of conjugation chemistry for POC diagnostics, offering improved performance and accuracy for rapid and reliable disease diagnosis.

## 2. Results and Discussion

The successful development of effective POC diagnostics relies on the use of optimized conjugation methods that enable the selective and controlled conjugation of antibodies to the assay platform [32,33]. This can significantly improve the sensitivity and specificity of immunoassays, enabling accurate and reliable disease diagnosis at the POC [1,34]. Over the past decade, there has been a significant advancement in engineering PEG chemistry [19,35]. A majority of current applications for PEG include therapeutic and diagnostic [16,17]. PEGylation of antibodies, in particular, has been known to increase the half-life and reduce the non-specific binding of antibodies, which hence leads to the economical production and usage of immunoassays [36,37]. Here, we describe a protocol for the site-directed conjugation of a bifunctional 20 kDa PEG linker to the Fc region of polyclonal anti-KPC-2 antibody to assist binding to gold screen-printed electrodes (Au-SPEs), one of the most common platforms used for electrochemical-based immunoassays at the POC [38,39,40]. This approach resulted in a packed conjugation of antibody molecules on the surface of Au-SPE, which is more favorable as there are more functional moieties to bind the antigen molecule. This also achieved enhanced capture of the KPC-2 antibody, supporting the potential for sensitive detection of antimicrobial resistance (AMR) caused by carbapenemases.

**Figure 1:**
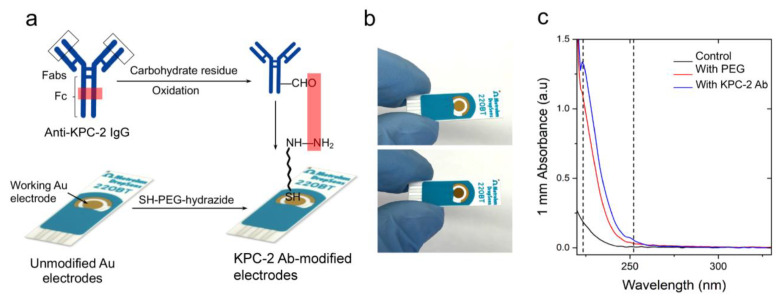
**PEG-mediated conjugation of IgG antibodies to the surface of screen-printed gold electrodes.** (**a**) Schematic presentation of the protocol for chemical modification of Au-SPE with SH-PEG-hydrazide followed by coupling with an antibody. (**b**) Digital images of unmodified (**top**) and modified Au-SPE (**bottom**), showing a change in color of the surface post-modification. (**c**) UV-Vis spectrum of control Au-SPE (without any treatment; black line), PEGylated Au-SPE (red line), and antibody-coupled Au-SPE (blue line). The surface of Au-SPEs was digested with TCEP, and the eluted solution was tested using a NanoDrop spectrophotometer (Thermofisher Scientific Inc, Waltham, MA, USA) to measure the absorbance.

To develop screen-printed electrodes for the detection of KPC-2 enzyme at POC, we designed a site-directed conjugation chemistry that relies on a heterobifunctional PEG polymer that links oxidized polyclonal anti-KPC-2 antibodies to the surface of Au-SPEs [41,42] (Figure 1a). PEGylation of the Au-SPE was conducted using thiolated-PEG-hydrazide (SH-PEG-hydrazide), which forms a non-covalent gold-thiol bond, leaving the PEG-hydrazide arm free to react [43]. The anti-KPC-2 polyclonal antibody was oxidized using sodium metaperiodate to convert the carbohydrate group in glycoproteins to reactive aldehyde groups. The free hydrazide group on the surface of Au-SPE reacted with the aldehyde group on the antibody Fc region for its directional conjugation (Figure 1a).

**Figure 2:**
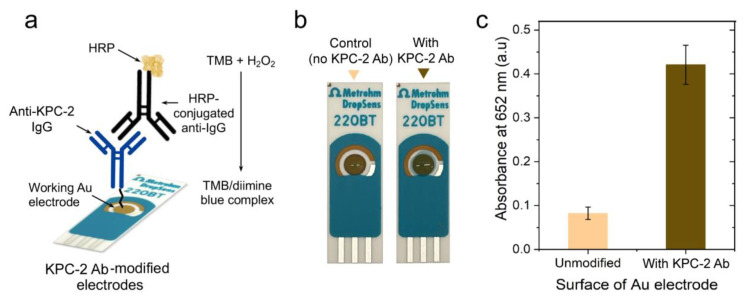
**Characterization of antibody-modified Au-SPEs using HRP-based colorimetry.** (**a**) Schematic presentation of the HRP-based colorimetry used to characterize the surface of the modified electrodes. The unmodified and modified Au-SPEs were incubated with HRP-conjugated anti-IgG and washed. Aliquots of TMB (10 µL) substrate were added to each strip and incubated for 10 min. (**b**) Digital images captured for the strips after TMB addition, showing a color change of TMB suggesting the presence of HRP. (**c**) The UV-Vis absorbance of TMB collected from the surface of electrodes was measured at 652 nm.

**Figure 3:**
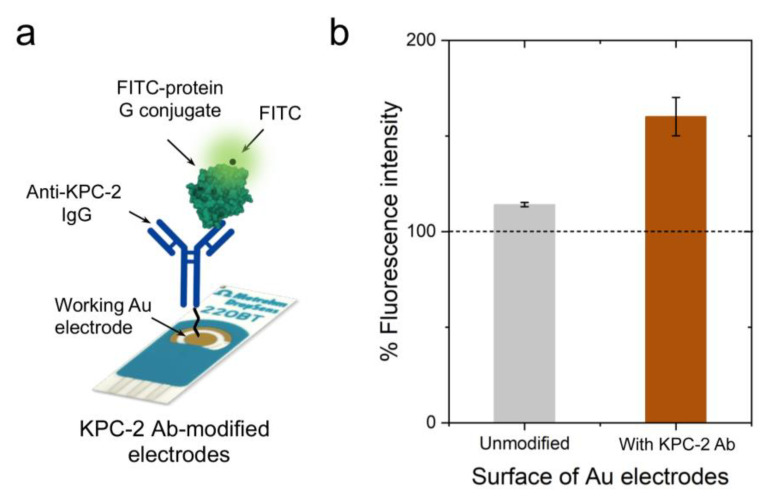
**Characterization of antibody-modified Au-SPEs using FITC-based fluorometry.** (**a**) Schematic presentation of the FITC-based staining protocol for Au-SPE characterization. The antibody-coated Au-SPEs were incubated with protein G coupled with FITC, which reacts with the antibody present on the surface of the electrode. (**b**) The surface-modified and unmodified electrodes were digested with TCEP to release the coupled antibody stained with protein G-FITC. Fluorescence intensity was measured for the released analyte at 528 nm.

To evaluate the efficiency of the developed PEG-based site-directed conjugation approach, we tested the presence of antibodies on the surface of Au-SPEs using multiple techniques, including colorimetry, fluorometry, Ultraviolet-visible (UV-Vis), Fourier-transform infrared (FTIR) spectroscopy, and sodium dodecyl-sulfate polyacrylamide gel electrophoresis (SDS-PAGE) analysis [44]. The digital images captured for the unmodified (i.e., no PEG and/or antibody) and PEG/antibody-modified Au-SPEs showed a change in the color of the modified electrode surface (Figure 1b). In addition, the Au-SPEs were digested using tris (2-carboxyethyl) phosphine (TCEP), and the UV-Vis spectrophotometry technique was used to confirm the presence of antibodies in the eluted solution [45,46,47]. The characteristic absorbance peak for PEG was observed near 220 nm, and the absorbance peak for the antibody was observed near 260 nm, compared with unmodified control Au-SPEs (Figure 2c). To further characterize the anti-KPC-2 IgG antibody-modified Au-SPEs, a horseradish peroxidase (HRP)-based colorimetry technique was used. The unmodified and anti-KPC-2 IgG-modified electrodes were incubated with HRP-conjugated anti-IgG for 15 min, and then they were gently washed three times, and then 10 µL of 3,3′,5,5′-tetramethylbenzidine (TMB) substrate was added to the Au-SPEs (Figure 2a). The complex was incubated with TMB for 10 min, and a strong blue color was seen in the antibody-modified SPE. This suggests that the HRP-bound anti-IgG was able to successfully bind the anti-KPC-2 antibody and was able to reduce the TMB to produce a blue color. The TMB added to the unmodified SPE did not produce any color change, indicating the absence of anti-KPC-2 antibodies. Further, the reduced TMB substrate was collected, and the absorbance of the formed complex was measured at 652 nm. The sharp rise in the absorbance at 652 nm confirmed the presence of anti-KPC-2 antibodies on the surface of Au-SPE and its labeling with HRP. The KPC-2 antibody-modified Au-SPEs were also incubated with protein-G coupled with fluorescein isothiocyanate (FITC) for 15 min (Figure 3a). Protein G tends to bind to most IgG molecules at near physiological pH at room temperature (25 °C). The bound antibody complex was then digested using TCEP from both unmodified and modified SPEs to release the IgG-protein G-FITC complex from the surface of Au-SPE, and the fluorescence signal was measured for the released complex at 528 nm. The coupled FITC to protein G was able to produce a fluorescent signal at 528 nm, which was ~68.2% higher than the control sample of unmodified electrodes, indicating a successful conjugation of antibody to the surface of Au-SPE.

**Figure 4:**
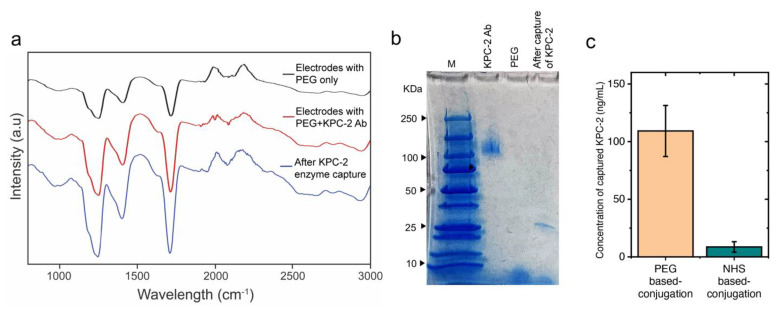
**Characterization of antibody-modified Au-SPEs and KPC−2 enzyme capture efficiency.** (**a**) FTIR analysis of the unmodified electrodes (in black), modified (in red), and modified electrodes after enzyme capture (in blue) post-TCEP digestion (**b**) SDS-PAGE analysis of modified with anti-KPC-2, unmodified, and modified with anti-KPC-2 with enzyme captured electrodes after TCEP digestion, showing the presence of bands specific to antigen at 27.2 KDa. (**c**) The concentration of KPC-2 enzyme captured on the surface of Au-SPEs modified with KPC-2 antibodies using the developed PEG-based conjugation compared with the traditional NHS-based conjugation chemistry. The surface of Au-SPEs (5 electrodes) was modified with antibody, loaded with 10 µL of KPC-2 enzyme at a concentration of 20 ng/mL, and incubated for 30 min at room temperature. After incubation, the unbound target enzyme was collected, and the concentration of the captured target KPC-2 enzyme was estimated from the total concentration of the loaded KPC-2 enzyme using UV-Vis spectroscopy.

To test the efficiency of the antibody-modified SPEs to capture the target KPC-2 enzyme, antibody-modified Au-SPEs were used to capture carbapenemases (at a very low concentration of 20 ng/mL). The SPEs were incubated with the KPC-2 β-lactamase for 30 min and washed 3 times using 10 mM phosphate buffer pH 7.2 to remove excess unbound enzyme. The complex was then digested using TCEP, and the released complex was used to perform SDS-PAGE and FTIR analyses. FTIR analysis results showed absorption bands of the amide I group at 1625 cm^−1^ (C−O stretching vibration of peptide linkages) and the COOH group (C=O stretching vibration) at around 1784.05 cm^−1^ (Figure 4a), indicating a successful surface modification with antibody and enzyme capture using the modified Au-SPEs [48,49,50]. The TCEP-digested complex from the electrodes’ surface was also analyzed using the SDS-PAGE technique. The presence of a band specific to KPC-2 antigen at 27.2 KDa in the TCEP digested sample indicates successful and effective capture of the KPC-2 β-lactamase by the antibody coated on the surface of Au-SPE. In addition, we used UV-Vis spectroscopy to compare the capture efficiency of KPC-2 enzyme on the surface of Au-SPE modified using the developed PEG protocol with the traditional NHS-based chemistry that relies on using succinimidyl 3-(2-pyridyldithio)propionate (SPDP). The NHS ester reacts with the amine groups of anti-KPC-2 IgG antibodies, and the pyridyldithiol reactive groups in the generated SPDP-modified antibody molecules bind to the surface of Au-SPEs. The results indicated a 12.6 ± 2.1 fold increase in the capture of the target KPC-2 enzyme using our PEG-based site-specific direct conjugation approach (Figure 4c).

PEGylation of antibodies affects their affinity for their target receptor. PEG functionalization of antibodies is also known to modify the biological activity of antibodies. Hence, optimal PEGylation (depending on the arm number or arm length of PEG) is necessary as it controls the sensitivity of the antigen-antibody interaction system. For instance, in non-exclusive target recognition by diagnostic probes, it becomes crucial to enhance specific targets to achieve a distinct contrast from the control. In such a case, PEGylation of antibodies has been shown to enhance antigen/ target recognition. A conditional aptamer that was synthesized by conjugating PEG5000-azobenzene-NHS, which was responsive to hypoxia and in turn created a ‘caging moiety’ causing a conditional recognition of the target [51]. The PEG moiety also endows a ‘stealth effect’ on the attached antibody, enabling selective targeting of antigen-overexpressing tissues by the antibody [52]. Moreover, the application of the PEG chemistry for detection of cancer-related antigens using screen-printed electrodes was reported much earlier and was a highly sensitive immunosensor with a sensitivity as low as 2.38 pg/mL, thus making the technique of directional conjugation widely accepted [53].

In this study, we presented a novel conjugation chemistry for developing enhanced KPC-2 enzyme testing at POC settings using SPEs-based electrochemical immunoassay [54]. Our approach relied on a site-directed reaction that used a heterobifunctional PEG polymer to link oxidized polyclonal anti-KPC-2 antibodies to the surface of Au-SPEs. We achieved PEGylation of the Au-SPE using SH-PEG-hydrazide, which left the hydrazide groups free to react with the oxidized Fc regions of anti-KPC-2 polyclonal antibodies. Our results demonstrated that this site-directed conjugation approach offered a viable alternative to traditional NHS-based chemistry for the modification of Au-SPEs with antibodies. By using directional conjugation, we were able to obtain highly efficient and specific binding of the antibody to the enzyme, which is crucial for designing and developing accurate detection of KPC-2. We also conducted a series of tests to confirm the presence of the antibody on the surface of the modified Au-SPEs, including colorimetry, fluorometry, UV-Vis and FTIR spectroscopy, and SDS-PAGE analysis. Our findings demonstrated the successful capture of the KPC-2 enzyme using the modified Au-SPEs, with the antibody-modified Au-SPEs capturing the enzyme at a very low concentration of 20 ng/mL. These promising results may have implications for the development of diagnostic tools for the early detection of infections caused by KPC-2 or other carbapenemase-producing bacteria, which is crucial for the effective management and control of antibiotic-resistant infections. Overall, our study highlights the potential of our PEG-based site-directed conjugation approach for the development of sensitive and specific POC diagnostics for a range of diseases and conditions.

## 3. Methods

**Au-SPE surface activation with PEG**. The surface of Au-SPEs is cleaned using a 75% ethanol solution for 5 min. The cleaned electrodes were washed with Milli-Q water and allowed to dry at room temperature (25 °C). Freshly prepared PEG solution (20 mg/mL; thiol-PEG-hydrazide) was drop-cast on the surface of the working electrode of Au-SPE and incubated for 1 h. After the reaction was completed, the modified electrodes were washed with water three times and used for the antibody coupling reaction.

**Antibody oxidation and coupling to Au-SPE**. Aliquots of 20 µL of antibody (1.2 mg/mL) were mixed with 10 mM sodium metaperiodate (pH = 5.5), acidified with 0.1 M sodium acetate buffer (pH = 5.5), and incubated at 4 °C in the dark for 20 min. Post-oxidation, the antibody was washed with phosphate buffer (pH = 7.4) using centrifugal filter units with a cut-off value of 50 kDa. The oxidized antibody solution was incubated with the PEG-modified Au-SPE.

**Characterization of antibody-modified electrodes**. The surface modification of Au-SPEs was characterized using UV-Vis spectroscopy. The modified and unmodified SPEs were digested with TCEP (three to five SPEs per test), and the protein concentration in the eluted solution was measured using a NanoDrop 2000/2000c Spectrophotometer (Thermo Fisher Scientific, Inc., Waltham, USA). To perform the HRP-based colorimetric assay, the antibody-modified and unmodified SPEs were incubated with HRP-conjugated anti-IgG antibodies for 20 min and washed three times gently. 10 µL of TMB substrate was added to each electrode and incubated for 10 min. A blue color was developed after the incubation period. The absorbance intensity of the formed blue color was also measured at 652 nm. The unmodified and modified SPEs were incubated with protein G coupled with FITC to bind the IgG on the surface of the electrode. After 15 min of incubation, the SPEs were washed gently three times to remove excess unbound protein G. The electrode surface was then digested with TCEP to release the bound protein G with FITC, and the fluorescence intensity of the released solution was measured at 528 nm to confirm the binding of Protein G to the antibodies on the surface of the electrodes.

**Enzyme capture assay and assessment of enzyme capture efficiency**. We incubated antibody-coupled SPEs and PEG-modified SPEs (no antibody) with the target KPC-2 enzyme (10 mg/mL). The surface of each electrode was washed and treated with a 100 mg/mL TCEP solution to release the bound protein. UV-Vis and Fourier transform-infrared (FTIR) spectroscopy techniques were performed to confirm the enzyme capture.

**Ultraviolet-visible (UV-Vis) spectroscopy**. Absorption spectra were measured on the NanoDrop 2000/2000c Spectrophotometer (Thermo Fisher Scientific, Inc., USA). Concentration was calculated by measuring the absorbance of 1 µL of the sample at 260/280 nm at room temperature. Each sample was tested 3–5 times, and average values are presented.

**Fourier transform infrared (FTIR) spectroscopy**. FTIR spectra in the region of 500–2000 cm^−1^ were collected in absorbance mode with a FTS 135 BIO-RAD FTIR spectrometer.

**SDS gel electrophoresis**. Protein testing was performed using SDS-PAGE on a 20% Tris-Glycine gel. The collected KPC-2 antibody and captured enzyme aliquots were digested in digesting buffer and heated for five minutes at 95 °C on a heat block. For gel electrophoresis analysis, 12 μL of a protein standard and 35 μL of the centrifuged samples were loaded on the gel using a mannoprotein electrophoresis apparatus (Bio-Rad, Hercules, CA, USA). The samples were electrophoresed at 90 V for 50 min. After electrophoresis was conducted, the gel was rinsed in water for 3 min. Then the gel was stained with the Biosafe Coomassie blue stain for about 1 h. Finally, the gel was de-stained and photographed.

## Data Availability

Data is contained within the article.
